# Proteomic and functional data sets on synaptic mitochondria from rats with genetic ablation of *Parkin*

**DOI:** 10.1016/j.dib.2018.08.053

**Published:** 2018-08-28

**Authors:** Lance M. Villeneuve, Kelly L. Stauch, Phillip S. Purnell, Howard S. Fox

**Affiliations:** Department of Pharmacology and Experimental Neuroscience, University of Nebraska Medical Center, Omaha, NE 68198, United States

## Abstract

In this paper, we provide proteomic and functional data for synaptic mitochondria from the striatum of rats with *Parkin* ablation. The quantitative proteomic data was obtained using SWATH-MS methodology and mitochondrial function was assessed through measurement of oxygen consumption rate using the Seahorse XF Analyzer. This data facilitates comparisons with previous proteomic and functional data obtained using the exact same methods. A complete set of proteomic data is contained in Supplementary Table 1.

**Specifications Table**

**Table:** 

Subject area	Biology
More specific subject area	Neurobiology
Type of data	Table, Graph
How data was acquired	SWATH-MS: TripleTOF 5600 (SCIEX), Respiration: Seahorse XF24 Extracellular Flux Analyzer
Data format	Analyzed
Experimental factors	Genetic ablation of *Park2* (Parkin)
Experimental features	Striatal synaptic mitochondria were isolated from 3-month-old male Parkin KO rats and age-matched controls and used for respiration studies or proteins were processed for mass spectrometry.
Data source location	Omaha, NE
Data accessibility	The Seahorse data are included in this article. The complete SWATH-MS data can be accessed in Supplementary Table 1.

**Value of the data**

• These data sets provide a useful resource to identify different synaptic mitochondrial processes that are affected due to loss of Parkin.
• This data will aid in comparisons of synaptic mitochondrial changes in Parkin KO rats with other PD animal models.

## 1 Data

**Fig. 1:**
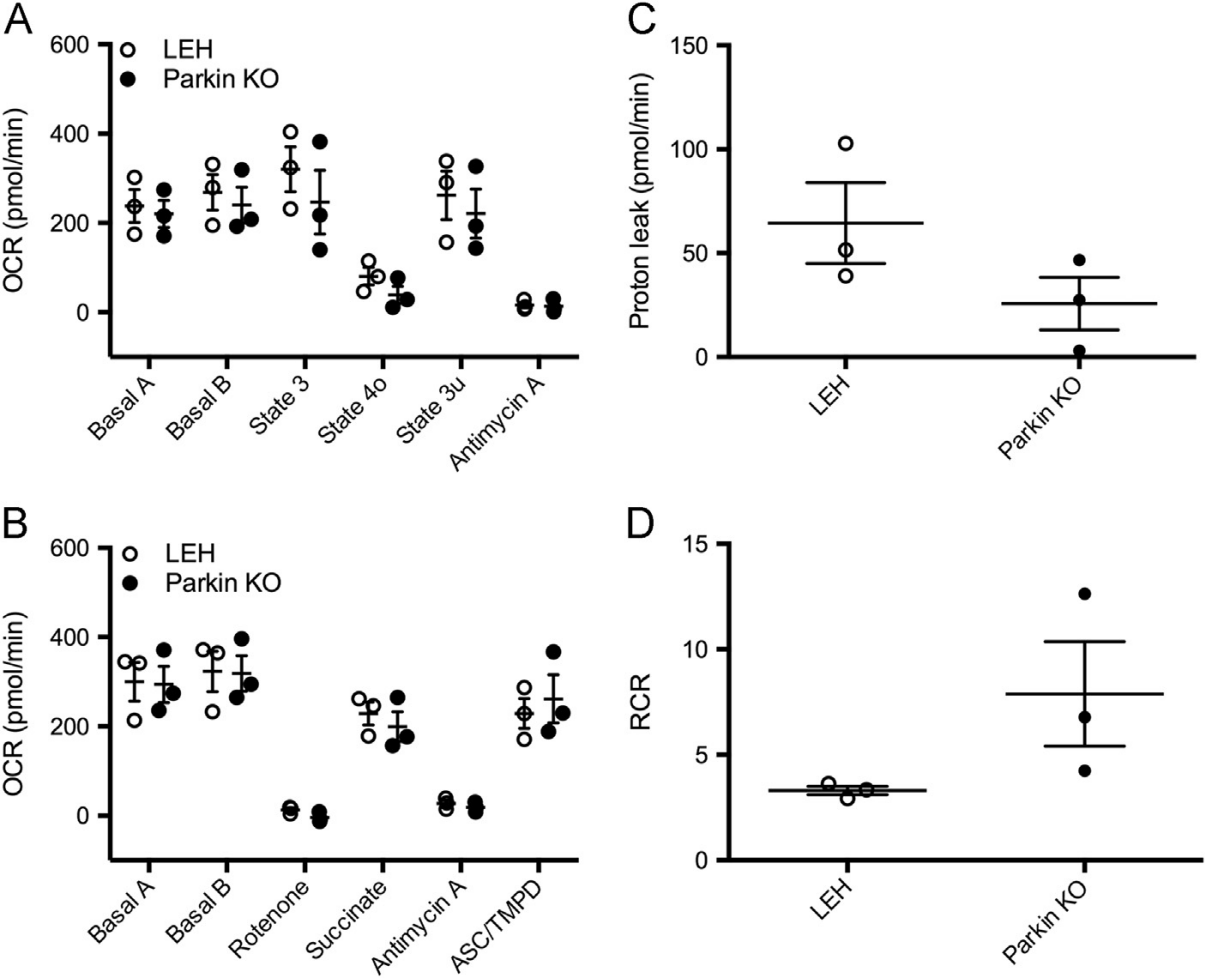
Striatal synaptic mitochondrial bioenergetics. (A) Mitochondrial state 2 (basal), state 3 (ADP-stimulated), state 4o (leak), and state 3 u (uncoupled) oxygen consumption rates (OCR) were measured in striatal synaptic mitochondria from 3-month-old male Parkin KO and age-matched controls (*n* = 3). (B) Subunits of the electron transport chain were interrogated using various toxins with basal being complex I-dependent respiration, succinate being complex II-dependent respiration, and ASC/TMPD being complex IV-dependent respiration. (C) Proton leak was calculated from state 4o minus antimycin A (Fig. 1C). (D) RCR was calculated using state 3 u/state 4o.

Bioenergetic data generated using the Seahorse XF^e^96 Extracellular Flux Analyzer is provided. No significant alteration of either the respiratory state (Fig. 1A) or the electron transport chain function (Fig. 1B) for striatal synaptic mitochondria from 3-month-old male Parkin KO rats was found. Furthermore, no significant alteration was present in either the amount of proton leak (Fig. 1C) or the respiratory control ratio (RCR, Fig. 1D), which is an overall measure of mitochondrial health.

SWATH-MS-based proteomic data is presented for striatal synaptic mitochondria from 3-month-old Parkin KO rats, compared to control animals. The list of 131 differentially expressed proteins is provided in Supplementary Table 1. Consistently changed proteins found from the comparison of the striatal synaptic and non-synaptic mitochondria [1] isolated from Parkin KO rats are shown in Table 1. Furthermore, common differentially expressed proteins in striatal synaptic mitochondria from Parkin KO and PINK1 KO rats [2] are shown in Table 2.

**Table 1:** Differentially expressed proteins in both striatal non-synaptic and synaptic mitochondria from Parkin KO compared to control rats. Protein expression values listed are log_2_ (Parkin KO/LEH). List of striatal non-synaptic mitochondrial proteins significantly altered in Parkin KO rats obtained from previously published work [1].

UniProt	Protein	Gene	Non-synaptic	Synaptic
P04636	Malate dehydrogenase	Mdh2	−1.253	−3.126
P30904	Macrophage migration inhibitory factor	Mif	−3.819	−3.759
P63081	V-type proton ATPase 16 kDa proteolipid subunit	Atp6v0c	2.107	−2.190

**Table 2:** Differentially expressed proteins in striatal synaptic mitochondria from both Pink1 KO and Parkin KO compared to control rats. Protein expression values listed are log_2_ (KO/LEH). List of striatal synaptic mitochondrial proteins significantly altered in PINK1 KO rats obtained from previously published work [2].

UniProt	Protein	Gene	Pink1 KO/LEH	Parkin KO/LEH
P03889	NADH-ubiquinone oxidoreductase chain 1	Mtnd1	−1.118	−2.151
P11884	Aldehyde dehydrogenase	Aldh2	−0.825	−1.279
P12007	Isovaleryl-CoA dehydrogenase	Ivd	−0.848	−2.947
P15650	Long-chain specific acyl-CoA dehydrogenase	Acadl	−1.004	−1.179
P20070	NADH-cytochrome b5 reductase 3	Cyb5r3	−1.270	−1.879
P22062	Protein-L-isoaspartate(D-aspartate) O-methyltransferase	Pcmt1	−1.009	−1.612
P38718	Mitochondrial pyruvate carrier 2	Mpc2	−1.188	−1.702
P51650	Succinate-semialdehyde dehydrogenase	Aldh5a1	−0.854	−2.582
P52504	NADH dehydrogenase [ubiquinone] iron-sulfur protein 6	Ndufs6	1.176	−5.979
P63031	Mitochondrial pyruvate carrier 1	Mpc1	0.905	−2.777
Q64536	[Pyruvate dehydrogenase (acetyl-transferring)] kinase isozyme 2	Pdk2	−0.955	−1.557
Q68FU3	Electron transfer flavoprotein subunit beta	Etfb	−1.424	−2.352
Q6B345	Protein S100-A11	S100a11	1.256	−2.990
Q75Q41	Mitochondrial import receptor subunit TOM22 homolog	Tomm22	−1.435	−2.144

## 2 Experimental design, materials and methods

### 2.1 Animals

Male Long Evans Hooded (LEH) (RRID:RGD_2308852) control and Parkin KO rats [3] were used at 3 months of age (three rats from each strain for respiration studies, *n* = 3; and four rats from each strain for proteomic experiments, *n* = 4). All protocols were conducted within NIH-approved guidelines for the Care and Use of Laboratory Animals with the approval and oversight of the University of Nebraska Medical Center Institutional Animal Care and Use Committee.

### 2.2 Isolation of synaptic mitochondria and respiration analysis

Brains were rapidly isolated from the animals, and the striatum (identified as per [4]) was removed by an investigator blinded to rat genotype and immediately rinsed with ice-cold 1x Phosphate Buffered Saline (Sigma, 806552) to remove blood. Tissue was chopped and homogenized using 10 strokes with a Dounce homogenizer (abcam, ab110169). Striatal synaptic mitochondria were isolated as previously described [5] with slight modifications [6] and oxygen consumption rates were measured using 3–4 technical replicate wells (7.5 μg of striatal synaptic mitochondria per well) for each biological replicate with a Seahorse XFe24 Analyzer (Agilent) for the previously described coupling and electron flow assays [7]. For data calculation the Seahorse Wave software (v2.2.0) was used. Prism (GraphPad) was used for graphs and statistical analyses (ANOVA and Sidak׳s multiple comparisons post-hoc testing).

### 2.3 Sample preparation for mass spectrometry

Synaptic mitochondria were lysed in 4% sodium dodecyl sulfate (Gibco, 15553), protein concentration was determined using a Pierce 660 nm Protein Assay (Thermo Fisher Scientific, 22660), and the filter aided sample preparation method [8] was used to prepare the synaptic mitochondrial peptides for mass spectrometry as described previously [9].

### 2.4 Data-independent SWATH-MS analysis

Striatal synaptic mitochondrial peptides were analyzed using SWATH data-independent analysis (DIA) mode on a TripleTOF 5600 (SCIEX) followed by targeted data extraction as described previously [10] with PeakView software (v2.1, SCIEX, **RRID:SCR_015786**) using our published reference spectral library [2], [9], [11]. CyberT (http://cybert.ics.uci.edu/) [12] was used with a sliding window of 61 and a Bayesian confidence coefficient of 12 and proteins were denoted as significantly altered following multiple-hypothesis testing correction if Benjamini & Hochberg (BH) *q*-values < 0.05.

## Funding

Funding support was provided by a grant from the Michael J Fox Foundation (Grant #9524).

## Appendix A Supplementary material

Supplementary Table 1. SWATH-MS quantitative proteomic data set for striatal synaptic mitochondria from 3-month-old male Parkin KO and control rats. Proteins were denoted as differentially expressed following multiple-hypothesis testing correction if Benjamini & Hochberg (BH) q-values < 0.05, highlighted in red.
